# 2017 ACC/AHA Blood Pressure Classification and Cardiovascular Disease in 15 Million Adults of Age 20–94 Years

**DOI:** 10.3390/jcm8111832

**Published:** 2019-11-01

**Authors:** Hokyou Lee, So Mi Jemma Cho, Jong Heon Park, Sungha Park, Hyeon Chang Kim

**Affiliations:** 1 Department of Preventive Medicine, Yonsei University College of Medicine, Seoul 03722, Korea; hokyou.lee@yuhs.ac; 2 Department of Internal Medicine, Yonsei University College of Medicine, Seoul 03722, Korea; shpark0530@yuhs.ac; 3 Department of Public Health, Yonsei University Graduate School, Seoul 03722, Korea; jemmasomicho@yuhs.ac; 4 Big Data Steering Department, National Health Insurance Service, Wonju 26464, Korea; parkjh@nhis.or.kr; 5 Division of Cardiology, Severance Cardiovascular Hospital and Cardiovascular Research Institute, Yonsei University College of Medicine, Seoul 03722, Korea

**Keywords:** hypertension, guideline, age-specific risk, population attributable risk, real world data

## Abstract

The 2017 American College of Cardiology/American Heart Association (ACC/AHA) high blood pressure (BP) guideline lowered the cut-off for hypertension, but its age-specific association with cardiovascular disease (CVD) remains inconclusive in different populations. We evaluated the association between high BP according to the 2017 ACC/AHA guideline and CVD risks in Koreans aged 20–94 years. In a nationwide health screening cohort, we included 15,508,537 persons aged 20–94 years without prior CVD. BP was categorized into normal, elevated, stage 1 hypertension, or stage 2 hypertension. The primary outcome was a composite CVD hospitalization (myocardial infarction, stroke, and/or heart failure). Over 10 years of follow-up, CVD incidence rates per 100,000 person-years were 105.4, 168.3, 215.9, and 641.2 for normal, elevated BP, stage 1, and stage 2 hypertension, respectively. The age-specific hazard ratios of stage 1 hypertension compared to normal BP were 1.41 (1.34–1.48) at ages 20–34, 1.54 (1.51–1.57) at ages 35–49, 1.38 (1.35–1.40) at ages 50–64, 1.21 (1.19–1.24) at ages 65–79, and 1.11 (1.03–1.19) at ages 80–94 years. With the lowered BP cut-off, 130/80 mmHg, population attributable fraction for CVD was 32.2%. In conclusion, stage 1 hypertension was significantly associated with a higher CVD risk across entire adulthood. The new definition of hypertension may have a substantial population impact on primary CVD prevention.

## 1. Introduction

The American College of Cardiology (ACC) and the American Heart Association (AHA) released the 2017 guideline for the management of high blood pressure (BP), with a lowered definition of hypertension as systolic blood pressure (SBP) ≥130 mmHg or diastolic blood pressure (DBP) ≥80 mmHg [1]. With the new definition, the prevalence of hypertension is expected to increase, although only a fraction of those newly identified would be recommended for pharmacologic treatment [2,3,4]. The decision for initiating antihypertensive medications in stage 1 hypertension is based on a 10-year atherosclerotic cardiovascular disease (ASCVD) risk calculation by the ACC/AHA Pooled Cohort Equations [5]. That said, the Pooled Cohort Equations were developed and validated for non-Hispanic white and African Americans aged 40 to 79 years [5], and it is still unknown whether the new guideline is applicable to other populations, and whether its impact is consistent across different ages.

Recent observational studies on long-term cardiovascular outcomes in Asian population according to the 2017 ACC/AHA BP category reported significant associations between stage 1 hypertension and incident cardiovascular disease (CVD) among young and middle-aged adults [6,7]. On the contrary, in older individuals, the association between stage 1 hypertension and CVD risk was inconclusive [7,8], and data on ages above 80 years are not available. We therefore evaluated the age-specific CVD risks associated with high BP according to the 2017 ACC/AHA guideline in Korean adults aged 20 to 94 years.

## 2. Materials and Methods

### 2.1. Data Source

We used the National Health Insurance Service (NHIS) database, which includes de-identified claim records of the entire Korean population. The NHIS is the single provider of universal healthcare coverage in South Korea, with two insurance types: a health insurance program covering 97% of the population and a medical aid program covering the remaining 3% with financial needs or under special provisions (e.g., national meritorious persons). The NHIS database contains demographics, hospital claims with International Classification of Disease, 10th edition (ICD-10) coding, death information, and health check-up results [9]. A description of the data source had been done in previous studies [10,11]. The study protocol was approved by the Institutional Review Board of Yonsei University Health System, Seoul, Korea (approval #Y-2019-0081). Informed consent was not required, as this is a retrospective study of de-identified administrative data.

### 2.2. Study Population

We identified 17,470,949 persons, aged 20 to 94 years, who had undergone nationwide general health screening provided by the NHIS from 1 January 2003 to 31 December 2007. We included 16,562,950 individuals with complete health examination results and sociodemographic information (excluding medical aid). We excluded persons with previous myocardial infarction (MI), stroke, or heart failure (N = 886,895) and those with less than one year of follow-up (N = 167,518). The final 15,508,537 persons were followed from the last examination before 31 December 2007 until death, migration from database, or 10 years after follow-up started, whichever came first (Figure S1).

### 2.3. Health Examination and Variables

The NHIS provides a general health screening program to all Korean adults every 1–2 years. The participation rate for the general health screening was 77.7% in 2016 [12]. The health screening centers are designated and quality controlled according to the relevant national laws and regulations; details of the examination are described elsewhere [13]. The main exposure was BP, categorized into normal (SBP <120 mmHg and DBP <80 mmHg), elevated (SBP 120–129 mmHg and DBP <80 mmHg), stage 1 hypertension (SBP 130–139 mmHg or DBP 80–89 mmHg), and stage 2 hypertension (SBP ≥140 mmHg or DBP ≥90 mmHg), according to the 2017 ACC/AHA guideline [1]. The BP measurement protocol recommended at least 5 min of rest in a seated position followed by ≥2 BP measurements by either auscultatory or oscillometric methods [14]. Persons prescribed an antihypertensive medication within one year prior to the examination were identified from the claims database [10,15] and grouped separately. Concurrent uses of glucose- or lipid-lowering medications [16] and Charlson comorbidity index [17,18] were also determined using insurance claims within the year prior to the examination. Information on smoking status (never, past, or current), alcohol consumption (none, 1–2 times/week, or ≥3 times/week), and physical exercise (none, 1–2 times/week, or ≥3 times/week) were self-reported, and body mass index (BMI) (kg/m^2^), fasting glucose (mg/dL), and total cholesterol (mg/dL) levels were collected during the examinations.

### 2.4. Outcomes

The primary outcome was a composite CVD event, defined as the first hospitalization for MI (ICD-10, I21–I23), stroke (ICD-10, I60–I64), and/or heart failure (ICD-10, I50), as done in previous studies [6,11]. The secondary outcome was all-cause death. Deaths were ascertained by linkage to the national registry via resident registration numbers. Further endpoint-specific analyses assessed MI, stroke, heart failure, and CVD-related death as separate outcomes—that is, if multiple types of event occurred, the first event of each type was counted separately. A CVD-related death was defined as mortality during or following hospitalization with ICD-10 codes I00–I99 within 30 days.

### 2.5. Statistical Analyses

Baseline characteristics were reported as mean ± standard deviation, median and interquartile range, or frequency and percent. CVD incidence and mortality rates per 100,000 person-years were calculated for each BP group. Cox proportional-hazards model was used to calculate hazard ratio (HR) and 95% confidence interval (CI) adjusted for age, sex, household income quartile, Charlson comorbidity index, use of glucose-lowering drugs, use of lipid-lowering drugs, tobacco smoking, alcohol consumption, physical exercise, BMI, fasting glucose, and total cholesterol. The proportional-hazards assumption was not violated according to graphical inspection of log-minus-log plot and Schoenfeld residuals. We additionally calculated HRs according to continuous SBP and DBP in penalized spline terms [19] to minimize possible bias from misclassification or terminal digit preference. We also stratified our analyses by sex, to evaluate sex-specific incidence rates and relative hazards for CVD and mortality. Furthermore, we performed the following four sensitivity analyses. First, among persons not taking an antihypertensive medication, we excluded those who started the medication within one year after the examination (remaining N = 12,908,843), given that the exposure to high BP may change upon initiation of the antihypertensive medication. Second, we merged those taking an antihypertensive medication with stage 2 hypertension, given that the threshold for treatment in the Korean Society of Hypertension guideline was SBP ≥140 mmHg or DBP ≥90 mmHg [20,21]. Third, we re-categorized BP across all health examinees, regardless of the antihypertensive medication. Fourth, to further minimize misclassification from single examination, we selected 7,536,232 persons, who underwent ≥2 examinations from 2003 through 2007 while not taking any antihypertensive medication and assigned them to the BP groups when the two most recent measurements met the corresponding cut-offs. All analyses were performed using SAS version 9.4 (SAS Institute Inc., Cary, NC, USA) and R version 3.4.4 (R Foundation for Statistical Computing, Vienna, Austria).

## 3. Results

### 3.1. Baseline Characteristics

A total of 15,508,537 individuals were followed for a median of 10 years. Baseline characteristics of each age group are summarized in Table 1. The proportion of women was 46.2% and increased with age (42.9% in 20–39 years to 54.5% in 80–94 years). Among all health examinees, 4,720,627 (30.4%) had stage 1 hypertension, 2,036,741 (13.1%) had stage 2 hypertension, and 2,048,253 (13.2%) were taking antihypertensive medications. Baseline characteristics by BP category were reported for each age group in Supplementary Materials (Tables S1–S5). At young and middle ages, 20–64 years, persons with untreated hypertension were more likely to have unhealthy lifestyles—such as tobacco smoking, frequent drinking, and less physical activity—despite having higher BMI, than those without hypertension (Tables S1–S3). At ages 65–94 years, persons with hypertension but not taking antihypertensive medications were less likely to have concurrent glucose-lowering or lipid-lowering medications, despite having higher fasting glucose and total cholesterol levels, than those without hypertension (Tables S4 and S5). Follow-up durations were comparable between the BP groups.

### 3.2. Primary Analysis

**Figure 1:**
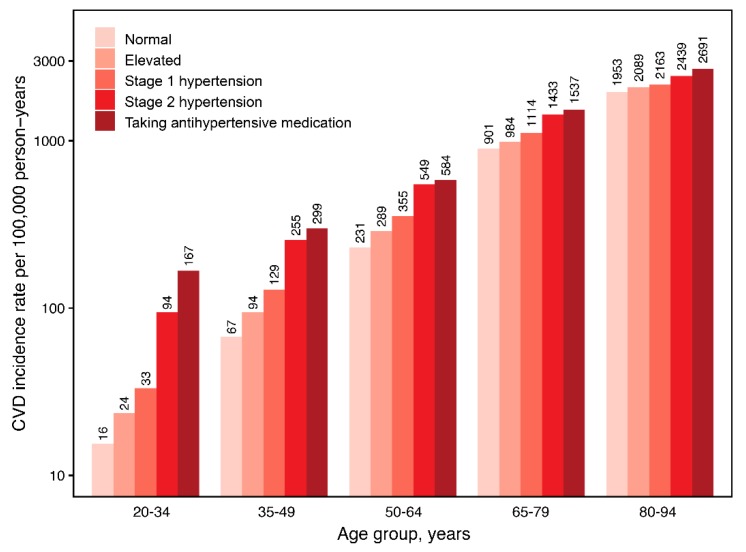
Age-specific CVD incidence rate according to blood pressure group. Rate per 100,000 person-years. CVD, cardiovascular disease.

During 151,285,314 person-years, 422,681 CVD events and 704,933 deaths occurred. Among persons not taking antihypertensive medications, CVD incidence rates per 100,000 person-years were 105.4, 168.3, 215.9, and 641.2, and mortality rates were 226.7, 310.6, 360.7, and 962.8 for normal, elevated BP, stage 1, and stage 2 hypertension, respectively. Among persons taking antihypertensive medications, CVD incidence and mortality rates were 798.2 and 1173.4 per 100,000 person-years, respectively. When stratified by age, the absolute risks for CVD and mortality increased toward older age and higher BP category (Figure 1 and Figure S2).

**Figure 2:**
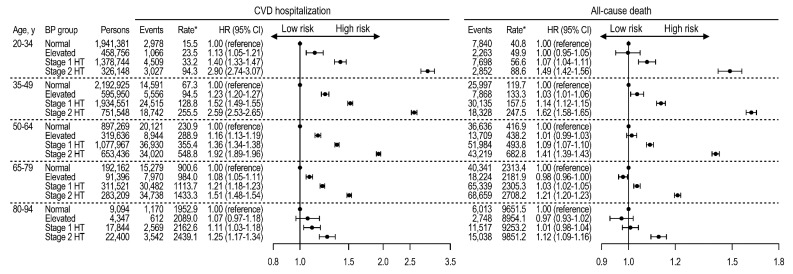
Age-specific CVD and mortality risks according to blood pressure group among persons not taking antihypertensive medications. Rates per 100,000 person-years. HRs adjusted for age, sex, household income, Charlson comorbidity index, use of glucose-lowering drugs, use of lipid-lowering drugs, smoking, drinking, exercise, body mass index, fasting glucose, and total cholesterol. BP, blood pressure; CI, confidence interval; CVD, cardiovascular disease; HR, hazard ratio; HT, hypertension.

Among persons not taking antihypertensive medications, stage 1 and stage 2 hypertension, with reference to normal BP, were significantly associated with increased CVD and mortality risks across a wide range of age, although the relative hazards by high BP attenuated with increasing age (Figure 2). For CVD events, when stage 1 hypertension was compared with normal BP, HR (95% CI) values were 1.40 (1.33–1.47) at ages 20–34 years, 1.52 (1.49–1.55) at ages 35–49 years, 1.36 (1.34–1.38) at ages 50–64 years, 1.21 (1.18–1.23) at ages 65–79 years, and 1.11 (1.03–1.18) at ages 80–94 years. When elevated BP was compared with normal, higher CVD risks were observed at ages 20 to 79 years—HR (95% CI), 1.13 (1.05–1.21) at ages 20–34 years, 1.23 (1.20–1.27) at ages 35–49 years, 1.16 (1.13–1.19) at ages 50–64 years, and 1.08 (1.05–1.11) at ages 65–79 years. The HRs for CVD events were unchanged by competing risks of death [22] (Table S6). For all-cause deaths, stage 1 and 2 hypertensions were significantly associated with higher mortality, compared with normal BP, at ages 20 to 64 years. However, at ages ≥80 years, the mortality risk by stage 1 hypertension was insignificant. Furthermore, in older individuals, the mortality risk followed a J-curve trend, such that persons with elevated BP had lower mortality risks than those with normal BP—HR (95% CI), 0.97 (0.96–0.99) at ages 65–79 years, and 0.97 (0.93–1.02) at ages 80–94 years—although by small differences.

**Figure 3:**
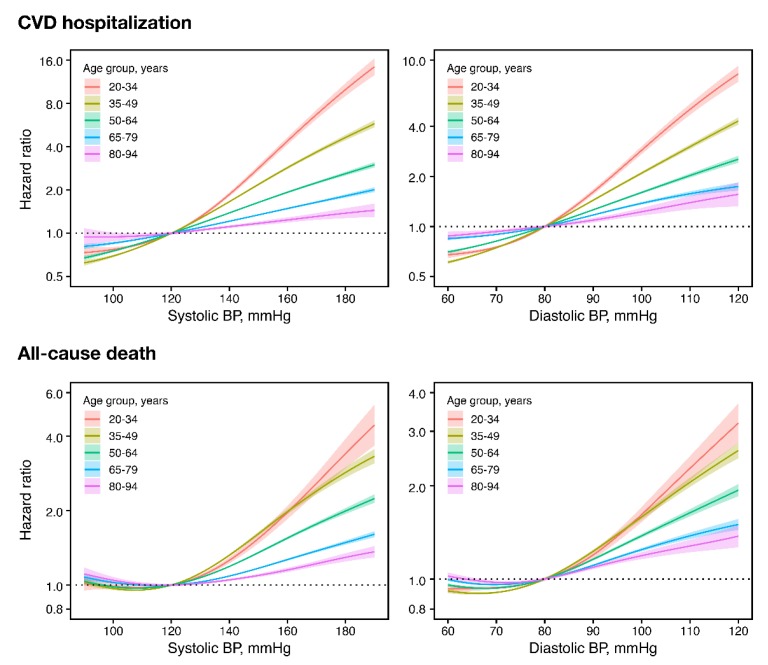
Age-specific CVD and mortality risks according to continuous systolic or diastolic BP among persons not taking antihypertensive medications. Hazard ratios in solid lines and 95% confidence intervals in shades. Hazard ratios centered on 120 mmHg systolic and 90 mmHg diastolic BP using penalized spline methods (df = 4), and adjusted for age, sex, household income, Charlson comorbidity index, use of glucose-lowering drugs, use of lipid-lowering drugs, smoking, drinking, exercise, body mass index, fasting glucose, and total cholesterol. BP, blood pressure; CVD, cardiovascular disease.

When continuous BP in spline terms was used instead of the 2017 ACC/AHA classification, higher BP—above SBP 120 mmHg or DBP 80 mmHg—was significantly associated with increasing CVD risk at all ages (Figure 3). On the other hand, for mortality, there was a J-curve trend, with increasing risk below SBP 120 mmHg or DBP 80 mmHg, in older individuals (Figure 3). Even so, there was a consistent trend of increasing mortality risk above SBP of 120 mmHg and DBP of 80 mmHg at all ages.

When further stratified by sex, the absolute risks for CVD and mortality were higher in men, but the trends of increasing absolute and relative risks by high BP were seen in both men and women at all ages (Figure S3). For CVD events, association with stage 1 hypertension was significant in both sexes at all ages, except for women at ages 80–94 years (Figure S4). In general, the relative hazards by high BP were larger in women before age 65 years, but larger in men afterwards, compared with the opposite sex. Similarly, for mortality, stage 1 hypertension was significantly associated with higher risk in both sexes at ages 20–79 years, with relative hazards by high BP larger in women before 34 years of age, and larger in men at older ages.

### 3.3. Endpoint-Specific Analyses

**Figure 4:**
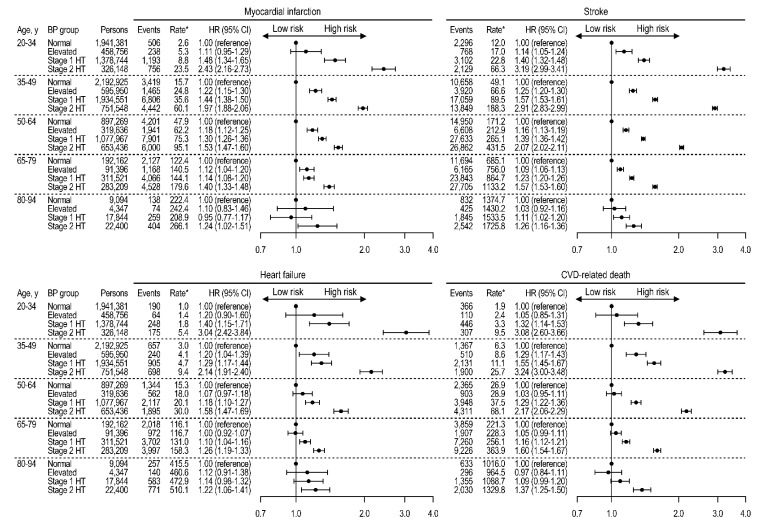
Age-specific risks for myocardial infarction, stroke, heart failure, and CVD-related deaths according to blood pressure group among persons not taking antihypertensive medications. Rates per 100,000 person-years. HRs adjusted for age, sex, household income, Charlson comorbidity index, use of glucose-lowering drugs, use of lipid-lowering drugs, smoking, drinking, exercise, body mass index, fasting glucose, and total cholesterol. BP, blood pressure; CI, confidence interval; CVD, cardiovascular disease; HR, hazard ratio; HT, hypertension.

In endpoint-specific analyses, MI, stroke, heart failure, and CVD-related deaths were assessed as separate outcomes (Figure 4). At ages 20 to 79 years, stage 1 hypertension was consistently associated with each CVD endpoint and CVD-related death. After age 80 years, stroke risk was significantly increased with stage 1 hypertension, while other outcomes were associated only with stage 2 hypertension.

### 3.4. Sensitivity Analyses

We further confirmed our findings in four sensitivity analyses. First, among persons not taking and have not started antihypertensive medications up to a year after examination (N = 12,908,843), we observed associations of BP groups with CVD and mortality comparable with those in the main analysis (Figure S5). We also found consistent results either when treated and stage 2 hypertensions were merged together (Figure S6), or when all examinees were re-categorized according to their measured BP regardless of antihypertensive medications (Figure S7). Finally, when we defined the BP groups using the two most recent examinations (N = 7,494,379, who underwent ≥2 examinations and were not taking any antihypertensive medication), stage 1 and 2 hypertensions were associated with increased hazards for CVD and mortality at all ages (Figure S8). The J-curve association between BP and mortality was less evident when multiple examinations were used.

## 4. Discussion

**Figure 5:**
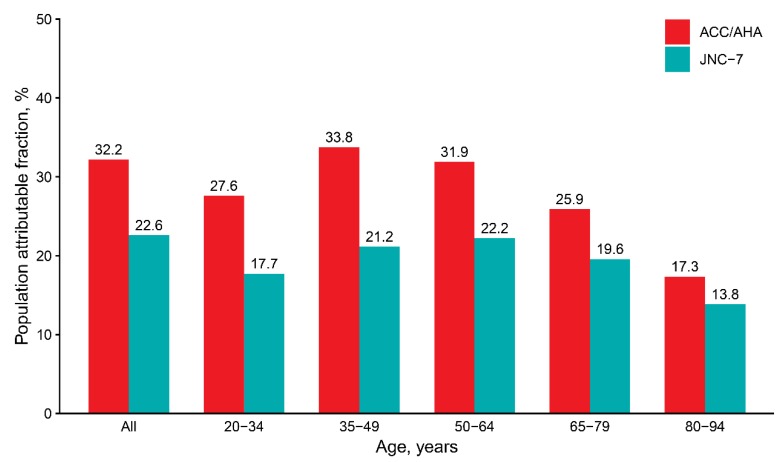
Age-specific population attributable fractions for cardiovascular disease associated with ACC/AHA or JNC-7 blood pressure cut-off. Adjusted hazard ratios (full model) were used for calculating population attributable fractions. ACC/AHA = SBP ≥130 mmHg, DBP ≥80 mmHg, or medication; JNC-7 = SBP ≥140 mmHg, DBP ≥90 mmHg, medication. ACC, American College of Cardiology; AHA, American Heart Association; DBP, diastolic blood pressure; JNC-7, Seventh Report of the Joint National Committee; SBP, systolic blood pressure.

In this nationwide study including Korean adults of wide age spectrum, we found significantly higher risks for CVD and mortality associated with elevated BP, stage 1, and stage 2 hypertensions according to the 2017 ACC/AHA guideline. Especially, stage 1 hypertension, of which the cut-off has been lowered by the new guideline, was associated with a significant increase of CVD risk throughout the entire adult ages. The population attributable fraction for CVD was 32.2% with the new definition of hypertension and 22.6% with the 2003 Seventh Report of the Joint National Committee (JNC-7) [23] cut-off (Figure 5).

The proportion of health examinees newly labeled by the 2017 ACC/AHA guideline, but not by JNC-7, as hypertension was particularly high among younger adults (33% among <50 years of age). However, the proportion of those newly recommended antihypertensive medications by the guideline may be small among younger adults [3]. Clinical benefits of pharmacological BP lowering were demonstrated mostly in older and high-risk individuals by randomized controlled trials, while the benefit-to-harm ratio in young adults may be less obvious [24]. In our study, the absolute risk for CVD in young adults taking antihypertensive medications was still as high as that in young adults with untreated stage 2 hypertension at baseline. However, it is noteworthy that young and middle-aged individuals with stage 1 hypertension, but not taking antihypertensive medications, had more risk factors and unhealthy lifestyles than those with normal BP in our study. Therefore, in young and middle-aged adults, the benefit of the lowered BP cut-offs may include the increased chance for lifestyle modifications and the reduction of lifetime CVD risk, as the effect of high BP and unhealthy lifestyles would sustain and accumulate over life in these age groups.

Conversely, in persons aged ≥65 years, average BPs were higher than in younger counterparts, and the relative CVD risk by high BP was less pronounced. Nevertheless, the absolute risk difference between those with and without stage 1 hypertension was substantial (220.8 per 100,000 person-years above 65 years of age). The benefit of intensive BP lowering in older or high-risk individuals was demonstrated in the Systolic Blood Pressure Intervention Trial (SPRINT) [25,26], as well as in meta-analyses [27]. Moreover, in our study, persons with untreated stage 1 or 2 hypertension at older ages (41% of persons aged ≥65 years) had more risk factors and poor metabolic profiles but were less likely to receive treatment or medical care for comorbid conditions, compared with those without hypertension. Therefore, in addition to the benefits from intensive BP lowering, the detection of stage 1 hypertension in the elderly may be valuable for comprehensive risk reduction and treatment for other comorbid conditions.

The associations between high BP and CVD risk had been previously established by a number of observational studies and pooled analyses including different populations [28,29,30,31,32,33,34]. However, since the release of the 2017 ACC/AHA guideline, the impact of the new BP classification in other populations is of new importance, as this guideline is likely to influence clinical practices in remaining parts of the world. Several recent studies on Asian population have tested the association between stage 1 hypertension and cardiovascular outcomes, but not for the entire adult ages. In young Korean adults, aged 20 to 39 years, Son et al. found a significantly higher CVD risk associated with elevated BP and stage 1 hypertension, compared with normal BP [6]. Another study by Qi et al. reported a significantly higher CVD risk associated with elevated BP and stage 1 hypertension among Chinese adults aged 35 to 59 years [7]. However, at ages ≥60 years, the association did not reach statistical significance [7]. In comparison, our study is the first to investigate the 2017 ACC/AHA BP classification on cardiovascular outcomes in Asian population over the entire adult ages, from 20 to 94 years. We found significant associations between stage 1 hypertension and CVD risk in the elderly population, even among ages of 80–94 years, to whom the Pooled Cohort Equations may not be applied. The 2017 ACC/AHA guideline extends the universal BP threshold, 130/80 mmHg, to patients of age ≥80 years, on the basis that these individuals would most likely have ≥10% 10-year ASCVD risks. Although our study demonstrated a significant CVD risk associated with stage 1 hypertension in this age group, the relative risk was modest and limited to stroke. Given the possibility of a J-curve relationship between BP and mortality, BP reduction in this age group should be sought with caution and individualized on biological age and functional status [35].

Our study has several strengths. We used a nationwide longitudinal database of all Korean adults participating in the national health screening program. With a high participation rate (77.7% in 2016), this database covers a wide range of Korean population over a long follow-up duration, and hence allows large numbers of participants and outcome events, detailed stratifications by age and sex, and especially, inclusion of participants aged ≥80 years, who are rarely studied in such large numbers. The outcome ascertainment rate is also high owing to electronic linkages to death records and universal insurance database. However, our study also has some limitations. First, given the retrospective and observational design, conclusions on treatment effect or target BP cannot be deduced from this study. The comparison of risks between the treated and untreated groups may be confounded by baseline risk features and local practice guidelines, which, in this case, recommend 140/90 mmHg as the threshold for treatment. Further research should address whether benefit can be reached for BP reduction initiated at stage 1 hypertension to a target BP <130/80 mmHg. Second, CVD events were obtained from the NHIS claims data, in which all medical information had been collected by healthcare professionals, but were not strictly adjudicated for research purpose; the operational definitions require further validations in future studies. Third, BP measurements and classification may be limited by single visits and lenient standardization. However, it is unlikely that these measurement errors would significantly distort the associations with outcomes. Furthermore, we repeated our analyses using multiple examinations or continuous BPs, all of which yielded consistent results. Fourth, some residual and unmeasured confounding factors, such as sodium intake, education level, and psychosocial factors, may exist. Finally, this study was based on Korean adults under a universal health insurance and screening program; the findings should thus be interpreted with caution when extended to different populations or unscreened individuals.

## 5. Conclusions

Among Korean adults aged 20 to 94 years, stage 1 hypertension was significantly associated with a higher CVD risk throughout the entire adult ages. Elevated BP was also associated with CVD at ages 20 to 79 years. The new definition of hypertension conveys a substantial population attributable risk, and thus may have a significant impact on the primary prevention of CVD at any ages.

## Figures and Tables

**Table 1:** Baseline characteristics by age group.

Variables				Age Group, Years		
	All	20–34	35–49	50–64	65–79	80–94
	(N = 15,508,537)	(N = 4,140,675)	(N = 5,894,433)	(N = 3,915,368)	(N = 1,466,247)	(N = 91,814)
Age, years	44 [34–55]	28 [25–31]	43 [39–46]	56 [53–60]	69 [67–73]	83 [81–85]
Sex						
Female	7,165,020 (46.2)	1,769,331 (42.7)	2,564,502 (43.5)	1,976,191 (50.5)	801,412 (54.7)	53,584 (58.4)
Male	8,343,517 (53.8)	2,371,344 (57.3)	3,329,931 (56.5)	1,939,177 (49.5)	664,835 (45.3)	38,230 (41.6)
Blood pressure group						
Normal	5,232,831 (33.7)	1,941,381 (46.9)	2,192,925 (37.2)	897,269 (22.9)	192,162 (13.1)	9094 (9.9)
Elevated	1,470,085 (9.5)	458,756 (11.1)	595,950 (10.1)	319,636 (8.2)	91,396 (6.2)	4347 (4.7)
Stage 1 hypertension	4,720,627 (30.4)	1,378,744 (33.3)	1,934,551 (32.8)	1,077,967 (27.5)	311,521 (21.2)	17,844 (19.4)
Stage 2 hypertension	2,036,741 (13.1)	326,148 (7.9)	751,548 (12.8)	653,436 (16.7)	283,209 (19.3)	22,400 (24.4)
Taking antihypertensive medication	2,048,253 (13.2)	35,646 (0.9)	419,459 (7.1)	967,060 (24.7)	587,959 (40.1)	38,129 (41.5)
Systolic blood pressure, mmHg	123.00 ± 16.26	117.53 ± 13.27	121.20 ± 15.04	127.35 ± 16.93	133.20 ± 18.29	136.61 ± 20.01
Diastolic blood pressure, mmHg	76.87 ± 10.67	73.83 ± 9.49	76.61 ± 10.64	79.22 ± 10.82	80.00 ± 10.98	80.64 ± 11.79
Body mass index, kg/m^2^	23.53 ± 3.19	22.62 ± 3.48	23.74 ± 3.00	24.17 ± 2.93	23.63 ± 3.19	22.21 ± 3.27
Fasting glucose, mg/dL	95.47 ± 25.35	88.77 ± 16.47	95.06 ± 24.09	100.32 ± 29.53	102.56 ± 32.34	104.44 ± 34.86
Total cholesterol, mg/dL	193.34 ± 36.95	181.48 ± 33.68	193.90 ± 35.66	202.52 ± 37.92	199.87 ± 39.06	195.98 ± 39.13
Taking glucose-lowering drugs	639,191 (4.1)	10,727 (0.3)	134,446 (2.3)	310,267 (7.9)	176,360 (12.0)	7391 (8.0)
Taking lipid-lowering drugs	653,191 (4.2)	19,462 (0.5)	162,284 (2.8)	332,781 (8.5)	134,869 (9.2)	3795 (4.1)
Charlson comorbidity index						
0	11,629,129 (75.0)	3,565,562 (86.1)	4,604,440 (78.1)	2,577,345 (65.8)	828,215 (56.5)	53,567 (58.3)
1	2,018,515 (13.0)	349,103 (8.4)	664,598 (11.3)	646,266 (16.5)	335,679 (22.9)	22,869 (24.9)
2	1,228,104 (7.9)	179,179 (4.3)	448,790 (7.6)	428,359 (10.9)	163,607 (11.2)	8169 (8.9)
≥3	632,789 (4.1)	46,831 (1.1)	176,605 (3.0)	263,398 (6.7)	138,746 (9.5)	7209 (7.9)
Tobacco smoking						
Never	10,205,344 (65.8)	2,453,754 (59.3)	3,699,455 (62.8)	2,833,911 (72.4)	1,143,800 (78.0)	74,424 (81.1)
Past	1,346,005 (8.7)	292,251 (7.1)	580,647 (9.9)	352,650 (9.0)	113,814 (7.8)	6643 (7.2)
Current	3,957,188 (25.5)	1,394,670 (33.7)	1,614,331 (27.4)	728,807 (18.6)	208,633 (14.2)	10,747 (11.7)
Alcohol consumption						
None	8,038,231 (51.8)	1,569,378 (37.9)	2,843,131 (48.2)	2,456,820 (62.7)	1,093,756 (74.6)	75,146 (81.8)
1–2/week	6,011,987 (38.8)	2,306,804 (55.7)	2,445,466 (41.5)	1,030,990 (26.3)	219,532 (15.0)	9195 (10.0)
≥3/week	1,458,319 (9.4)	264,493 (6.4)	605,836 (10.3)	427,558 (10.9)	152,959 (10.4)	7473 (8.1)
Physical exercise						
None	8,432,884 (54.4)	2,416,722 (58.4)	2,972,230 (50.4)	2,022,331 (51.7)	948,776 (64.7)	72,825 (79.3)
1–2/week	4,205,135 (27.1)	1,231,619 (29.7)	1,800,577 (30.5)	957,845 (24.5)	207,258 (14.1)	7836 (8.5)
≥3/week	2,870,518 (18.5)	492,334 (11.9)	1,121,626 (19.0)	935,192 (23.9)	310,213 (21.2)	11,153 (12.1)
Follow-up, years	10.0 [10.0–10.0]	10.0 [10.0–10.0]	10.0 [10.0–10.0]	10.0 [10.0–10.0]	10.0 [10.0–10.0]	7.6 [4.4–10.0]

Data are presented as mean ± standard deviation, median [interquartile range], or frequency (percent).

## Supplementary Materials

The following are available online at https://www.mdpi.com/2077-0383/8/11/1832/s1; Table S1: Baseline characteristics by blood pressure group at ages 20–34 years; Table S2: Baseline characteristics by blood pressure group at ages 35–49 years; Table S3: Baseline characteristics by blood pressure group at ages 50–64 years; Table S4: Baseline characteristics by blood pressure group at ages 65–79 years; Table S5: Baseline characteristics by blood pressure group at ages 80–94 years; Table S6: Age-specific CVD risks according to blood pressure group with or without competing risk analysis; Figure S1: Flowchart of inclusion and exclusion criteria; Figure S2: Age-specific mortality rate according to blood pressure group; Figure S3: Sex- and age-specific CVD incidence and mortality rates according to blood pressure group; Figure S4: Sex- and age-specific CVD and mortality risks according to blood pressure group among persons not taking antihypertensive medication; Figure S5: Age-specific CVD and mortality risks according to blood pressure group among persons who have not started antihypertensive medication up to one year of follow-up; Figure S6: Age-specific CVD and mortality risks according to blood pressure group with stage 2 hypertension defined as SBP ≥140 mmHg or DBP ≥90 mmHg or treated; Figure S7: Age-specific CVD and mortality risks according to blood pressure group with or without antihypertensive medication; Figure S8: Age-specific CVD and mortality risks according to blood pressure group defined by measurements from two different examinations.
