# Supplementary material for: 2017 ACC/AHA Blood Pressure Classification and Cardiovascular Disease in 15 Million Adults of Age 20–94 Years
Source: J Clin Med. 2019 Nov 1;8(11):1832. doi: 10.3390/jcm8111832 (PMC6912685; doi:10.3390/jcm8111832)
Supplement: Supplementary file 1 [file jcm-08-01832-s001.pdf]

Supplementary materials

Table S1. Baseline characteristics by blood pressure group at ages 20-34 years

Table S2. Baseline characteristics by blood pressure group at ages 35-49 years

Table S3. Baseline characteristics by blood pressure group at ages 50-64 years

Table S4. Baseline characteristics by blood pressure group at ages 65-79 years

Table S5. Baseline characteristics by blood pressure group at ages 80-94 years

Table S6. Age-specific CVD risks according to blood pressure group with or without competing risk analysis

Figure S1. Flowchart of inclusion and exclusion criteria

Figure S2. Age-specific mortality rate according to blood pressure group

Figure S3. Sex- and age-specific CVD incidence and mortality rates according to blood pressure group

Figure S4. Sex- and age-specific CVD and mortality risks according to blood pressure group among persons not taking antihypertensive medication

Figure S5. Age-specific CVD and mortality risks according to blood pressure group among persons who have not started antihypertensive medication up to one year of follow-up

Figure S6. Age-specific CVD and mortality risks according to blood pressure group with stage 2 hypertension defined as SBP ≥140 mmHg or DBP ≥90 mmHg or treated

Figure S7. Age-specific CVD and mortality risks according to blood pressure group with or without antihypertensive medication

Figure S8. Age-specific CVD and mortality risks according to blood pressure group defined by measurements from two different examinations

Table S1. Baseline characteristics by blood pressure group at ages 20-34 years

Variables	2017 ACC/AHA blood pressure group, untreated				Taking antihypertensive medication
	Normal (N=1,941,381)	Elevated (N=458,756)	Stage 1 hypertension (N=1,378,744)	Stage 2 hypertension (N=326,148)	(N=35,646)
Age, years	27 [25-31]	28 [25-31]	29 [26-32]	30 [27-32]	31 [28-33]
Sex					
Female	1,190,240 (61.3)	159,491 (34.8)	370,764 (26.9)	41,595 (12.8)	7,241 (20.3)
Male	751,141 (38.7)	299,265 (65.2)	1,007,980 (73.1)	284,553 (87.2)	28,405 (79.7)
Systolic blood pressure, mmHg	106.99 ± 7.33	122.30 ± 2.93	124.99 ± 7.75	140.43 ± 11.75	131.69 ± 18.00
Diastolic blood pressure, mmHg	66.97 ± 5.89	71.01 ± 4.36	80.14 ± 3.94	90.89 ± 8.15	83.44 ± 12.83
Body mass index, kg/m²	21.46 ± 2.92	22.83 ± 3.20	23.44 ± 3.48	25.37 ± 3.97	25.49 ± 4.33
Fasting glucose, mg/dL	86.80 ± 13.71	89.44 ± 15.53	89.89 ± 17.30	93.96 ± 23.50	97.21 ± 30.59
Total cholesterol, mg/dL	176.28 ± 31.66	181.00 ± 32.96	185.36 ± 34.18	195.39 ± 37.08	193.75 ± 38.36
Taking glucose-lowering drugs	2,593 (0.1)	854 (0.2)	3,613 (0.3)	1,446 (0.4)	2,221 (6.2)
Taking lipid-lowering drugs	4,174 (0.2)	1,600 (0.3)	6,411 (0.5)	2,262 (0.7)	5,015 (14.1)
Charlson Comorbidity Index					
0	1,663,217 (85.7)	397,081 (86.6)	1,197,370 (86.8)	286,685 (87.9)	21,209 (59.5)
1	178,668 (9.2)	37,360 (8.1)	106,361 (7.7)	21,652 (6.6)	5,062 (14.2)
2	78,645 (4.1)	19,417 (4.2)	60,119 (4.4)	14,390 (4.4)	6,608 (18.5)
≥3	20,851 (1.1)	4,898 (1.1)	14,894 (1.1)	3,421 (1.0)	2,767 (7.8)
Cigarette smoking					
Never	1,374,731 (70.8)	251,660 (54.9)	681,461 (49.4)	128,300 (39.3)	17,602 (49.4)
Past	109,056 (5.6)	35,734 (7.8)	112,752 (8.2)	30,482 (9.3)	4,227 (11.9)
Current	457,594 (23.6)	171,362 (37.4)	584,531 (42.4)	167,366 (51.3)	13,817 (38.8)
Alcohol consumption					
None	860,142 (44.3)	165,570 (36.1)	447,106 (32.4)	84,104 (25.8)	12,456 (34.9)
1-2/week	1,002,279 (51.6)	263,388 (57.4)	818,768 (59.4)	202,077 (62.0)	20,292 (56.9)
≥3/week	78,960 (4.1)	29,798 (6.5)	112,870 (8.2)	39,967 (12.3)	2,898 (8.1)
Physical exercise					
None	1,236,047 (63.7)	255,544 (55.7)	746,018 (54.1)	161,882 (49.6)	17,231 (48.3)
1-2/week	499,948 (25.8)	144,065 (31.4)	454,742 (33.0)	120,128 (36.8)	12,736 (35.7)
≥3/week	205,386 (10.6)	59,147 (12.9)	177,984 (12.9)	44,138 (13.5)	5,679 (15.9)
Household income, quartile					
Q4, highest	166,351 (8.6)	40,886 (8.9)	135,888 (9.9)	32,188 (9.9)	5,242 (14.7)
Q3	572,268 (29.5)	136,370 (29.7)	430,882 (31.3)	104,110 (31.9)	13,516 (37.9)
Q2	684,857 (35.3)	161,556 (35.2)	481,333 (34.9)	114,838 (35.2)	10,558 (29.6)
Q1, lowest	517,905 (26.7)	119,944 (26.1)	330,641 (24.0)	75,012 (23.0)	6,330 (17.8)
Follow-up, years	10.0 [10.0-10.0]	10.0 [10.0-10.0]	10.0 [10.0-10.0]	10.0 [10.0-10.0]	10.0 [10.0-10.0]

Data are presented as mean ± standard deviation, median [interquartile range], or frequency (percent).

Table S2. Baseline characteristics by blood pressure group at ages 35-49 years

Variables	2017 ACC/AHA blood pressure group, untreated				Taking antihypertensive medication (N=419,459)
	Normal (N=2,192,925)	Elevated (N=595,950)	Stage 1 hypertension (N=1,934,551)	Stage 2 hypertension (N=751,548)	
Age, years	43 [39-45]	43 [39-46]	42 [38-46]	43 [39-47]	45 [42-48]
Sex					
Female	1,259,890 (57.5)	263,482 (44.2)	661,064 (34.2)	211,471 (28.1)	168,595 (40.2)
Male	933,035 (42.5)	332,468 (55.8)	1,273,487 (65.8)	540,077 (71.9)	250,864 (59.8)
Systolic blood pressure, mmHg	107.45 ± 7.29	122.51 ± 2.95	125.53 ± 7.79	142.58 ± 12.83	132.96 ± 16.74
Diastolic blood pressure, mmHg	67.41 ± 5.92	71.83 ± 4.32	80.72 ± 3.93	92.28 ± 8.35	84.38 ± 11.42
Body mass index, kg/m²	22.78 ± 2.68	23.61 ± 2.79	24.03 ± 2.91	24.97 ± 3.13	25.40 ± 3.23
Fasting glucose, mg/dL	91.41 ± 19.13	94.32 ± 21.48	95.63 ± 24.29	100.15 ± 30.05	103.39 ± 32.59
Total cholesterol, mg/dL	188.27 ± 33.89	192.77 ± 34.78	196.36 ± 35.65	202.22 ± 37.71	198.70 ± 37.39
Taking glucose-lowering drugs	26,823 (1.2)	9,745 (1.6)	36,208 (1.9)	16,380 (2.2)	45,290 (10.8)
Taking lipid-lowering drugs	29,075 (1.3)	10,142 (1.7)	36,139 (1.9)	14,203 (1.9)	72,725 (17.3)
Charlson Comorbidity Index					
0	1,725,156 (78.7)	472,256 (79.2)	1,547,751 (80.0)	614,593 (81.8)	244,684 (58.3)
1	261,360 (11.9)	66,985 (11.2)	204,170 (10.6)	70,453 (9.4)	61,630 (14.7)
2	148,993 (6.8)	41,449 (7.0)	134,158 (6.9)	49,332 (6.6)	74,858 (17.8)
≥3	57,416 (2.6)	15,260 (2.6)	48,472 (2.5)	17,170 (2.3)	38,287 (9.1)
Cigarette smoking					
Never	1,556,370 (71.0)	378,657 (63.5)	1,103,827 (57.1)	396,777 (52.8)	263,824 (62.9)
Past	160,815 (7.3)	59,079 (9.9)	218,367 (11.3)	91,232 (12.1)	51,154 (12.2)
Current	475,740 (21.7)	158,214 (26.5)	612,357 (31.7)	263,539 (35.1)	104,481 (24.9)
Alcohol consumption					
None	1,242,778 (56.7)	295,489 (49.6)	833,597 (43.1)	278,643 (37.1)	192,624 (45.9)
1-2/week	807,293 (36.8)	246,958 (41.4)	872,962 (45.1)	344,325 (45.8)	173,928 (41.5)
≥3/week	142,854 (6.5)	53,503 (9.0)	227,992 (11.8)	128,580 (17.1)	52,907 (12.6)
Physical exercise					
None	1,166,243 (53.2)	298,144 (50.0)	949,985 (49.1)	368,002 (49.0)	189,856 (45.3)
1-2/week	617,542 (28.2)	182,071 (30.6)	621,391 (32.1)	245,779 (32.7)	133,794 (31.9)
≥3/week	409,140 (18.7)	115,735 (19.4)	363,175 (18.8)	137,767 (18.3)	95,809 (22.8)
Household income, quartile					
Q4, highest	877,025 (40.0)	236,120 (39.6)	747,806 (38.7)	269,170 (35.8)	166,482 (39.7)
Q3	551,471 (25.1)	157,014 (26.3)	531,655 (27.5)	212,718 (28.3)	108,186 (25.8)
Q2	380,447 (17.3)	103,798 (17.4)	342,161 (17.7)	142,351 (18.9)	74,476 (17.8)
Q1, lowest	383,982 (17.5)	99,018 (16.6)	312,929 (16.2)	127,309 (16.9)	70,315 (16.8)
Follow-up, years	10.0 [10.0-10.0]	10.0 [10.0-10.0]	10.0 [10.0-10.0]	10.0 [10.0-10.0]	10.0 [10.0-10.0]

Data are presented as mean ± standard deviation, median [interquartile range], or frequency (percent).

Table S3. Baseline characteristics by blood pressure group at ages 50-64 years

Variables	2017 ACC/AHA blood pressure group, untreated				Taking antihypertensive medication (N=967,060)
	Normal (N=897,269)	Elevated (N=319,636)	Stage 1 hypertension (N=1,077,967)	Stage 2 hypertension (N=653,436)	
Age, years	55 [52-59]	55 [53-59]	55 [53-59]	57 [53-61]	57 [53-61]
Sex					
Female	519,987 (58.0)	172,947 (54.1)	495,940 (46.0)	278,886 (42.7)	508,431 (52.6)
Male	377,282 (42.0)	146,689 (45.9)	582,027 (54.0)	374,550 (57.3)	458,629 (47.4)
Systolic blood pressure, mmHg	108.04 ± 7.31	122.75 ± 3.05	126.99 ± 7.59	146.11 ± 13.16	134.54 ± 16.87
Diastolic blood pressure, mmHg	67.78 ± 5.94	71.76 ± 4.41	80.44 ± 4.35	90.93 ± 8.69	83.03 ± 10.69
Body mass index, kg/m²	23.17 ± 2.69	23.76 ± 2.71	24.01 ± 2.79	24.50 ± 2.93	25.17 ± 2.99
Fasting glucose, mg/dL	95.27 ± 24.10	98.07 ± 26.47	99.02 ± 28.03	102.42 ± 32.06	105.80 ± 33.60
Total cholesterol, mg/dL	199.83 ± 36.76	202.26 ± 37.23	203.30 ± 37.58	205.92 ± 38.97	201.95 ± 38.63
Taking glucose-lowering drugs	39,868 (4.4)	17,960 (5.6)	57,096 (5.3)	33,685 (5.2)	161,658 (16.7)
Taking lipid-lowering drugs	41,176 (4.6)	16,301 (5.1)	49,632 (4.6)	24,339 (3.7)	201,333 (20.8)
Charlson Comorbidity Index					
0	613,870 (68.4)	219,133 (68.6)	756,151 (70.1)	478,387 (73.2)	509,804 (52.7)
1	146,811 (16.4)	52,401 (16.4)	166,425 (15.4)	92,985 (14.2)	187,644 (19.4)
2	86,729 (9.7)	30,760 (9.6)	100,095 (9.3)	53,792 (8.2)	156,983 (16.2)
≥3	49,859 (5.6)	17,342 (5.4)	55,296 (5.1)	28,272 (4.3)	112,629 (11.6)
Cigarette smoking					
Never	669,621 (74.6)	234,870 (73.5)	753,658 (69.9)	445,856 (68.2)	729,906 (75.5)
Past	65,245 (7.3)	27,575 (8.6)	103,551 (9.6)	65,144 (10.0)	91,135 (9.4)
Current	162,403 (18.1)	57,191 (17.9)	220,758 (20.5)	142,436 (21.8)	146,019 (15.1)
Alcohol consumption					
None	620,364 (69.1)	210,917 (66.0)	651,923 (60.5)	363,929 (55.7)	609,687 (63.0)
1-2/week	210,774 (23.5)	79,784 (25.0)	301,268 (27.9)	186,254 (28.5)	252,910 (26.2)
≥3/week	66,131 (7.4)	28,935 (9.1)	124,776 (11.6)	103,253 (15.8)	104,463 (10.8)
Physical exercise					
None	470,844 (52.5)	162,564 (50.9)	560,418 (52.0)	351,183 (53.7)	477,322 (49.4)
1-2/week	220,004 (24.5)	79,505 (24.9)	269,318 (25.0)	155,409 (23.8)	233,609 (24.2)
≥3/week	206,421 (23.0)	77,567 (24.3)	248,231 (23.0)	146,844 (22.5)	256,129 (26.5)
Household income, quartile					
Q4, highest	323,340 (36.0)	112,067 (35.1)	363,252 (33.7)	199,008 (30.5)	316,653 (32.7)
Q3	221,417 (24.7)	79,687 (24.9)	269,562 (25.0)	164,931 (25.2)	244,495 (25.3)
Q2	177,083 (19.7)	63,415 (19.8)	219,301 (20.3)	139,776 (21.4)	193,943 (20.1)
Q1, lowest	175,429 (19.6)	64,467 (20.2)	225,852 (21.0)	149,721 (22.9)	211,969 (21.9)
Follow-up, years	10.0 [10.0-10.0]	10.0 [10.0-10.0]	10.0 [10.0-10.0]	10.0 [10.0-10.0]	10.0 [10.0-10.0]

Data are presented as mean ± standard deviation, median [interquartile range], or frequency (percent).

Table S4. Baseline characteristics by blood pressure group at ages 65-79 years

Variables	2017 ACC/AHA blood pressure group, untreated				Taking antihypertensive medication (N=587,959)
	Normal (N=192,162)	Elevated (N=91,396)	Stage 1 hypertension (N=311,521)	Stage 2 hypertension (N=283,209)	
Age, years	69 [67-72]	69 [67-72]	69 [67-73]	69 [67-73]	69 [67-73]
Sex					
Female	101,836 (53.0)	47,717 (52.2)	159,132 (51.1)	144,433 (51.0)	348,294 (59.2)
Male	90,326 (47.0)	43,679 (47.8)	152,389 (48.9)	138,776 (49.0)	239,665 (40.8)
Systolic blood pressure, mmHg	108.59 ± 7.35	122.72 ± 3.13	128.37 ± 7.04	149.62 ± 13.64	137.52 ± 17.97
Diastolic blood pressure, mmHg	67.46 ± 6.23	70.44 ± 4.71	79.19 ± 5.12	88.69 ± 9.62	81.83 ± 10.98
Body mass index, kg/m²	22.29 ± 2.99	22.93 ± 2.96	23.13 ± 3.04	23.50 ± 3.13	24.50 ± 3.15
Fasting glucose, mg/dL	97.33 ± 27.61	99.43 ± 29.06	99.78 ± 29.90	101.97 ± 32.61	106.51 ± 34.82
Total cholesterol, mg/dL	194.94 ± 37.51	197.68 ± 37.99	199.22 ± 38.54	201.68 ± 39.55	201.30 ± 39.60
Taking glucose-lowering drugs	13,080 (6.8)	7,634 (8.4)	22,680 (7.3)	18,191 (6.4)	114,775 (19.5)
Taking lipid-lowering drugs	8,426 (4.4)	4,651 (5.1)	13,040 (4.2)	9,146 (3.2)	99,606 (16.9)
Charlson Comorbidity Index					
0	113,568 (59.1)	54,746 (59.9)	191,855 (61.6)	186,029 (65.7)	282,017 (48.0)
1	43,192 (22.5)	20,429 (22.4)	68,394 (22.0)	57,838 (20.4)	145,826 (24.8)
2	19,548 (10.2)	9,172 (10.0)	29,285 (9.4)	23,110 (8.2)	82,492 (14.0)
≥3	15,854 (8.3)	7,049 (7.7)	21,987 (7.1)	16,232 (5.7)	77,624 (13.2)
Cigarette smoking					
Never	142,280 (74.0)	68,873 (75.4)	235,921 (75.7)	214,767 (75.8)	481,959 (82.0)
Past	14,633 (7.6)	7,549 (8.3)	24,724 (7.9)	22,550 (8.0)	44,358 (7.5)
Current	35,249 (18.3)	14,974 (16.4)	50,876 (16.3)	45,892 (16.2)	61,642 (10.5)
Alcohol consumption					
None	147,566 (76.8)	68,554 (75.0)	227,948 (73.2)	199,138 (70.3)	450,550 (76.6)
1-2/week	27,283 (14.2)	13,853 (15.2)	48,202 (15.5)	44,865 (15.8)	85,329 (14.5)
≥3/week	17,313 (9.0)	8,989 (9.8)	35,371 (11.4)	39,206 (13.8)	52,080 (8.9)
Physical exercise					
None	125,339 (65.2)	57,005 (62.4)	207,416 (66.6)	193,210 (68.2)	365,806 (62.2)
1-2/week	28,646 (14.9)	13,714 (15.0)	42,448 (13.6)	35,977 (12.7)	86,473 (14.7)
≥3/week	38,177 (19.9)	20,677 (22.6)	61,657 (19.8)	54,022 (19.1)	135,680 (23.1)
Household income, quartile					
Q4, highest	79,370 (41.3)	38,444 (42.1)	125,931 (40.4)	105,474 (37.2)	249,357 (42.4)
Q3	45,579 (23.7)	21,228 (23.2)	74,071 (23.8)	66,581 (23.5)	133,029 (22.6)
Q2	32,752 (17.0)	14,931 (16.3)	52,680 (16.9)	51,480 (18.2)	94,299 (16.0)
Q1, lowest	34,461 (17.9)	16,793 (18.4)	58,839 (18.9)	59,674 (21.1)	111,274 (18.9)
Follow-up, years	10.0 [10.0-10.0]	10.0 [10.0-10.0]	10.0 [10.0-10.0]	10.0 [10.0-10.0]	10.0 [9.9-10.0]

Data are presented as mean ± standard deviation, median [interquartile range], or frequency (percent).

Table S5. Baseline characteristics by blood pressure group at ages 80-94 years

Variables	2017 ACC/AHA blood pressure group, untreated				Taking antihypertensive medication (N=38,129)
	Normal (N=9,094)	Elevated (N=4,347)	Stage 1 hypertension (N=17,844)	Stage 2 hypertension (N=22,400)	
Age, years	83 [81-85]	83 [81-85]	83 [81-85]	83 [81-85]	83 [81-84]
Sex					
Female	4,521 (49.7)	2,212 (50.9)	9,501 (53.2)	13,016 (58.1)	24,334 (63.8)
Male	4,573 (50.3)	2,135 (49.1)	8,343 (46.8)	9,384 (41.9)	13,795 (36.2)
Systolic blood pressure, mmHg	107.97 ± 7.74	122.37 ± 3.08	128.52 ± 6.79	152.33 ± 15.02	139.61 ± 19.79
Diastolic blood pressure, mmHg	66.81 ± 6.70	69.04 ± 5.08	78.61 ± 5.53	88.40 ± 10.64	81.65 ± 11.87
Body mass index, kg/m²	20.96 ± 3.01	21.51 ± 2.95	21.60 ± 3.11	21.99 ± 3.20	23.00 ± 3.28
Fasting glucose, mg/dL	100.29 ± 32.21	101.91 ± 35.59	101.63 ± 31.15	103.21 ± 33.90	107.75 ± 37.21
Total cholesterol, mg/dL	187.23 ± 36.86	190.23 ± 37.19	192.82 ± 38.45	196.12 ± 38.77	200.11 ± 39.83
Taking glucose-lowering drugs	366 (4.0)	221 (5.1)	700 (3.9)	774 (3.5)	5,330 (14.0)
Taking lipid-lowering drugs	112 (1.2)	78 (1.8)	244 (1.4)	228 (1.0)	3,133 (8.2)
Charlson Comorbidity Index					
0	5,430 (59.7)	2,722 (62.6)	11,297 (63.3)	15,301 (68.3)	18,817 (49.4)
1	2,331 (25.6)	1,054 (24.2)	4,270 (23.9)	4,806 (21.5)	10,408 (27.3)
2	704 (7.7)	315 (7.2)	1,274 (7.1)	1,359 (6.1)	4,517 (11.8)
≥3	629 (6.9)	256 (5.9)	1,003 (5.6)	934 (4.2)	4,387 (11.5)
Cigarette smoking					
Never	6,738 (74.1)	3,289 (75.7)	13,927 (78.0)	17,912 (80.0)	32,558 (85.4)
Past	781 (8.6)	371 (8.5)	1,376 (7.7)	1,573 (7.0)	2,542 (6.7)
Current	1,575 (17.3)	687 (15.8)	2,541 (14.2)	2,915 (13.0)	3,029 (7.9)
Alcohol consumption					
None	7,379 (81.1)	3,483 (80.1)	14,283 (80.0)	17,827 (79.6)	32,174 (84.4)
1-2/week	933 (10.3)	461 (10.6)	1,962 (11.0)	2,393 (10.7)	3,446 (9.0)
≥3/week	782 (8.6)	403 (9.3)	1,599 (9.0)	2,180 (9.7)	2,509 (6.6)
Physical exercise					
None	7,172 (78.9)	3,321 (76.4)	14,370 (80.5)	18,328 (81.8)	29,634 (77.7)
1-2/week	850 (9.3)	385 (8.9)	1,460 (8.2)	1,709 (7.6)	3,432 (9.0)
≥3/week	1,072 (11.8)	641 (14.7)	2,014 (11.3)	2,363 (10.5)	5,063 (13.3)
Household income, quartile					
Q4, highest	3,741 (41.1)	1,848 (42.5)	7,461 (41.8)	8,661 (38.7)	16,595 (43.5)
Q3	1,838 (20.2)	883 (20.3)	3,559 (19.9)	4,667 (20.8)	7,513 (19.7)
Q2	1,522 (16.7)	699 (16.1)	2,856 (16.0)	3,701 (16.5)	5,854 (15.4)
Q1, lowest	1,993 (21.9)	917 (21.1)	3,968 (22.2)	5,371 (24.0)	8,167 (21.4)
Follow-up, years	7.5 [4.2-10.0]	7.4 [4.1-10.0]	7.8 [4.5-10.0]	7.6 [4.4-10.0]	7.3 [4.2-10.0]

Data are presented as mean ± standard deviation, median [interquartile range], or frequency (percent).

Table S6. Age-specific CVD risks according to blood pressure group with or without competing risk analysis

Age, years	BP group	Persons	Events	HR (95% CI) for CVD hospitalization	
				Without competing risk	With competing risk*
20-34	Normal	1,941,381	2,978	1.00 (reference)	1.00 (reference)
	Elevated BP	458,756	1,066	1.13 (1.05-1.21)	1.13 (1.05-1.21)
	Stage 1 hypertension	1,378,744	4,509	1.40 (1.33-1.47)	1.40 (1.33-1.47)
	Stage 2 hypertension	326,148	3,027	2.90 (2.74-3.07)	2.89 (2.74-3.06)
35-49	Normal	2,192,925	14,591	1.00 (reference)	1.00 (reference)
	Elevated BP	595,950	5,556	1.23 (1.20-1.27)	1.23 (1.20-1.27)
	Stage 1 hypertension	1,934,551	24,515	1.52 (1.49-1.55)	1.52 (1.49-1.55)
	Stage 2 hypertension	751,548	18,742	2.59 (2.53-2.65)	2.58 (2.52-2.64)
50-64	Normal	897,269	20,121	1.00 (reference)	1.00 (reference)
	Elevated BP	319,636	8,944	1.16 (1.13-1.19)	1.16 (1.13-1.19)
	Stage 1 hypertension	1,077,967	36,930	1.36 (1.34-1.38)	1.36 (1.34-1.38)
	Stage 2 hypertension	653,436	34,020	1.92 (1.89-1.96)	1.90 (1.87-1.94)
65-79	Normal	192,162	15,279	1.00 (reference)	1.00 (reference)
	Elevated BP	91,396	7,970	1.08 (1.05-1.11)	1.09 (1.06-1.12)
	Stage 1 hypertension	311,521	30,482	1.21 (1.18-1.23)	1.21 (1.18-1.23)
	Stage 2 hypertension	283,209	34,738	1.51 (1.48-1.54)	1.48 (1.45-1.51)
80-94	Normal	9,094	1,170	1.00 (reference)	1.00 (reference)
	Elevated BP	4,347	612	1.07 (0.97-1.18)	1.08 (0.98-1.19)
	Stage 1 hypertension	17,844	2,569	1.11 (1.03-1.18)	1.10 (1.03-1.18)
	Stage 2 hypertension	22,400	3,542	1.25 (1.17-1.34)	1.21 (1.13-1.29)

*Death as a competing event to CVD by Fine-Gray model. Both models are adjusted for age, sex, household income, Charlson Comorbidity Index, use of glucose-lowering drugs, use of lipid-lowering drugs, smoking, drinking, exercise, body mass index, fasting glucose, and total cholesterol.
BP, blood pressure; CI, confidence interval; CVD, cardiovascular disease.

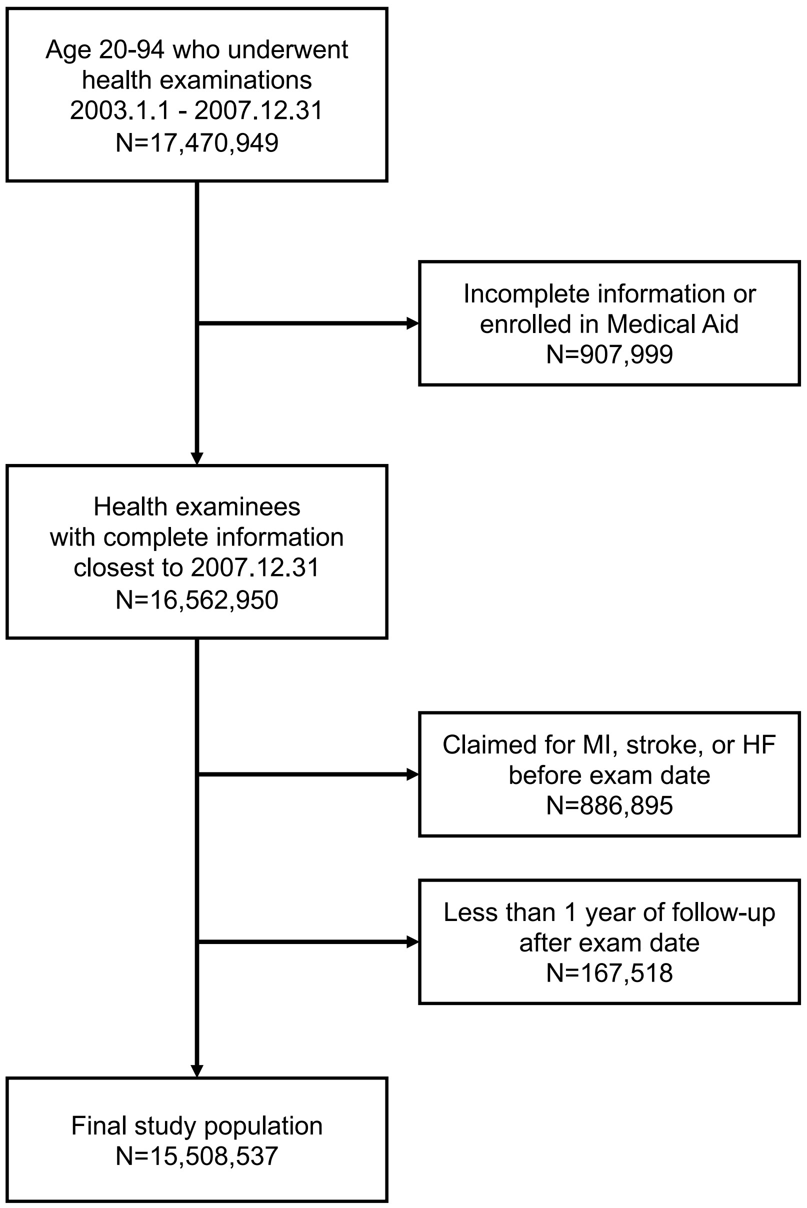

Figure S1. Flowchart of inclusion and exclusion criteria.
HF, heart failure; MI, myocardial infarction.

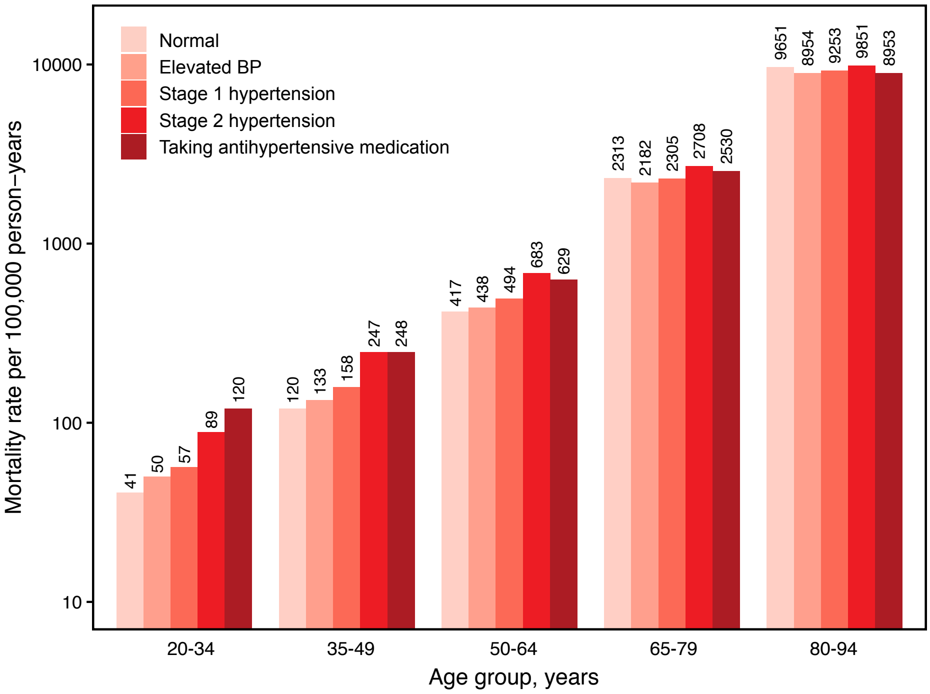

Figure S2. Age-specific mortality rate according to blood pressure group.

CVD hospitalization

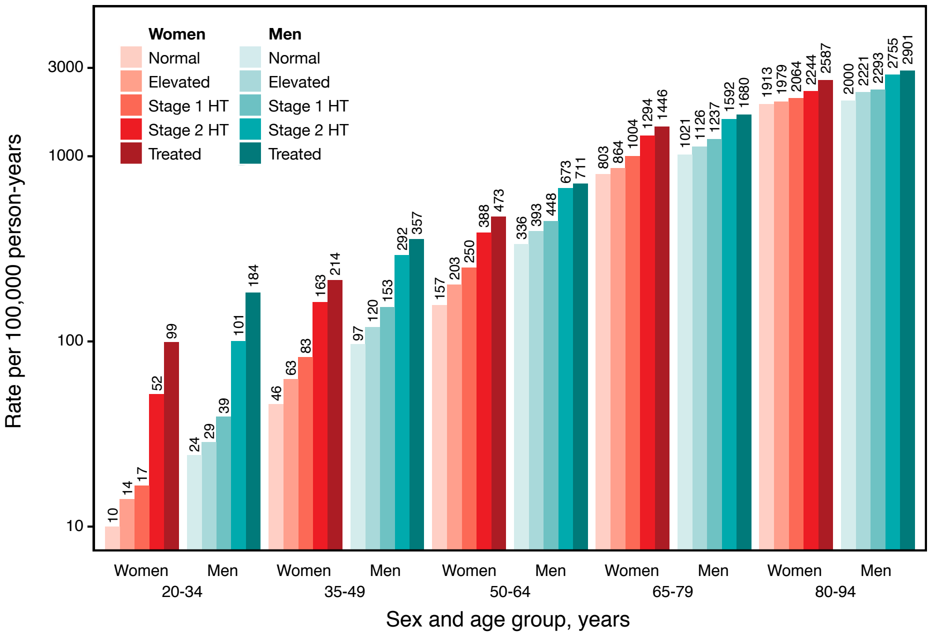

All-cause death

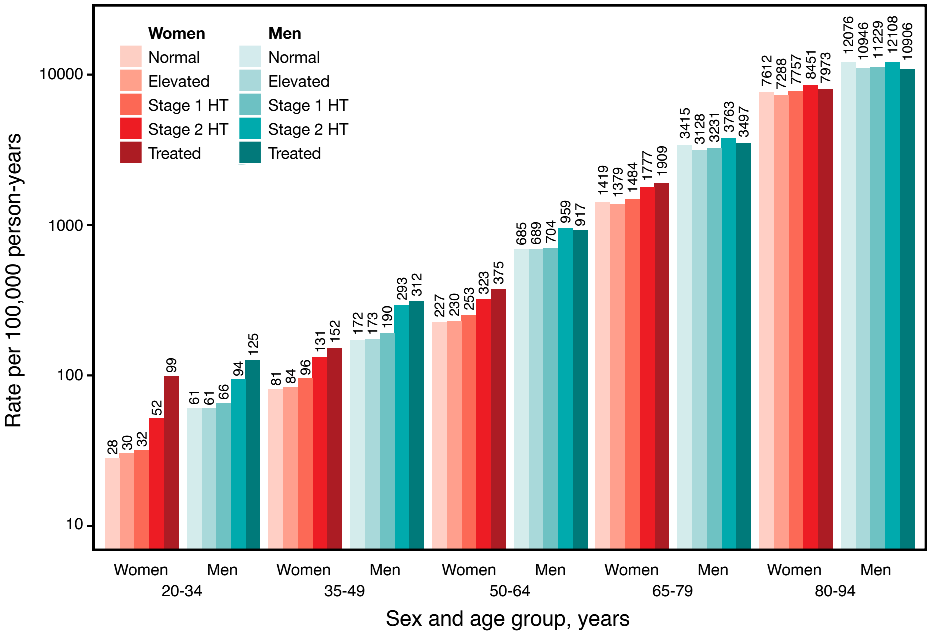

Figure S3. Sex- and age-specific CVD incidence and mortality rates according to blood pressure group. CVD, cardiovascular disease; HT, hypertension.

Men

Age, y	BP group	Persons	CVD hospitalization: Events	Rate*	HR (95% CI)	All-cause death: Events	Rate*	HR (95% CI)
20-34	Normal	751,141	1,792	24.3	1.00 (reference)	4,496	60.9	1.00 (reference)
	Elevated	299,265	843	28.6	1.08 (1.00-1.17)	1,784	60.5	0.98 (0.93-1.04)
	Stage 1 HT	1,007,980	3,896	39.4	1.36 (1.28-1.44)	6,520	65.8	1.07 (1.03-1.11)
	Stage 2 HT	284,553	2,813	100.6	2.75 (2.58-2.93)	2,639	94.1	1.48 (1.41-1.56)
35-49	Normal	933,035	8,864	96.5	1.00 (reference)	15,817	171.6	1.00 (reference)
	Elevated	332,468	3,916	119.7	1.22 (1.18-1.27)	5,678	172.9	1.06 (1.03-1.09)
	Stage 1 HT	1,273,487	19,114	152.9	1.50 (1.46-1.54)	23,843	189.7	1.16 (1.13-1.18)
	Stage 2 HT	540,077	15,337	292.0	2.53 (2.46-2.60)	15,573	293.3	1.67 (1.64-1.71)
50-64	Normal	377,282	12,103	335.5	1.00 (reference)	24,998	684.9	1.00 (reference)
	Elevated	146,689	5,507	393.4	1.14 (1.11-1.18)	9,779	688.7	1.03 (1.01-1.06)
	Stage 1 HT	582,027	24,806	447.9	1.31 (1.29-1.34)	39,616	703.7	1.10 (1.08-1.12)
	Stage 2 HT	374,550	23,516	673.0	1.86 (1.82-1.91)	34,357	958.7	1.45 (1.43-1.48)
65-79	Normal	90,326	7,743	1020.9	1.00 (reference)	26,689	3415.0	1.00 (reference)
	Elevated	43,679	4,172	1125.8	1.10 (1.06-1.14)	11,993	3128.1	0.98 (0.96-1.00)
	Stage 1 HT	152,389	15,885	1237.3	1.20 (1.17-1.23)	43,048	3230.7	1.03 (1.02-1.05)
	Stage 2 HT	138,776	18,031	1592.1	1.51 (1.47-1.55)	44,730	3763.4	1.22 (1.20-1.24)
80-94	Normal	4,573	549	2000.0	1.00 (reference)	3,437	12076.2	1.00 (reference)
	Elevated	2,135	296	2220.7	1.10 (0.96-1.27)	1,530	10946.6	0.98 (0.92-1.04)
	Stage 1 HT	8,343	1,173	2293.4	1.13 (1.02-1.25)	6,024	11228.5	0.99 (0.95-1.03)
	Stage 2 HT	9,384	1,529	2754.6	1.35 (1.22-1.49)	7,077	12108.2	1.10 (1.06-1.15)

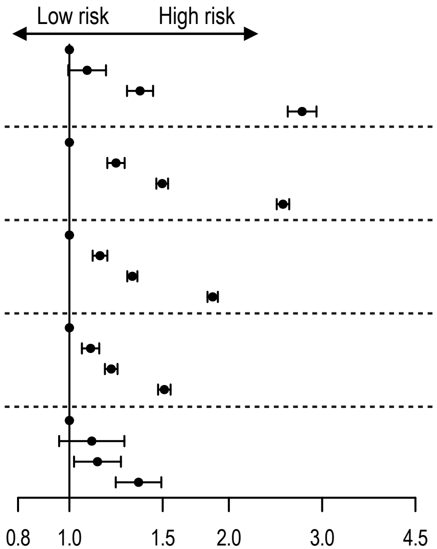

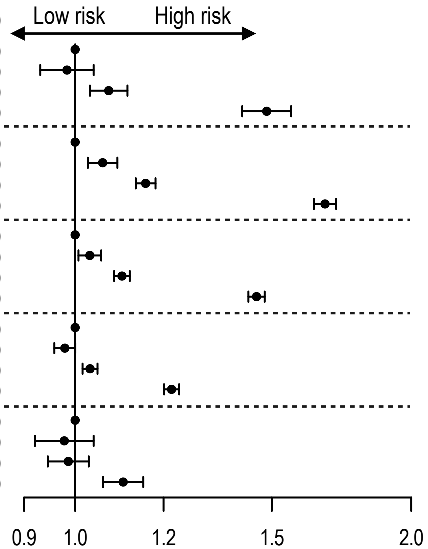

Women

Age, y	BP group	Persons	CVD hospitalization: Events	Rate*	HR (95% CI)	All-cause death: Events	Rate*	HR (95% CI)
20-34	Normal	1,190,240	1,186	10.0	1.00 (reference)	3,344	28.3	1.00 (reference)
	Elevated	159,491	223	14.1	1.30 (1.13-1.50)	479	30.2	1.04 (0.94-1.14)
	Stage 1 HT	370,764	613	16.7	1.50 (1.36-1.66)	1,178	32.0	1.08 (1.01-1.16)
	Stage 2 HT	41,595	214	51.9	3.83 (3.28-4.46)	213	51.6	1.60 (1.39-1.85)
35-49	Normal	1,259,890	5,727	45.8	1.00 (reference)	10,180	81.4	1.00 (reference)
	Elevated	263,482	1,640	62.8	1.27 (1.20-1.34)	2,190	83.7	0.99 (0.95-1.04)
	Stage 1 HT	661,064	5,401	82.6	1.62 (1.56-1.68)	6,292	95.9	1.12 (1.08-1.15)
	Stage 2 HT	211,471	3,405	163.4	2.86 (2.73-2.99)	2,755	131.4	1.44 (1.38-1.51)
50-64	Normal	519,987	8,018	157.0	1.00 (reference)	11,638	226.6	1.00 (reference)
	Elevated	172,947	3,437	202.7	1.19 (1.14-1.23)	3,930	230.0	0.97 (0.93-1.00)
	Stage 1 HT	495,940	12,124	249.9	1.43 (1.39-1.47)	12,368	252.6	1.05 (1.03-1.08)
	Stage 2 HT	278,886	10,504	388.3	2.01 (1.95-2.07)	8,862	322.7	1.27 (1.23-1.31)
65-79	Normal	101,836	7,536	803.3	1.00 (reference)	13,652	1418.8	1.00 (reference)
	Elevated	47,717	3,798	864.4	1.07 (1.02-1.11)	6,231	1379.0	0.98 (0.95-1.01)
	Stage 1 HT	159,132	14,597	1004.5	1.21 (1.18-1.25)	22,291	1484.2	1.04 (1.02-1.06)
	Stage 2 HT	144,433	16,707	1294.0	1.50 (1.46-1.54)	23,929	1776.9	1.19 (1.17-1.22)
80-94	Normal	4,521	621	1913.0	1.00 (reference)	2,576	7612.5	1.00 (reference)
	Elevated	2,212	316	1979.1	1.03 (0.90-1.18)	1,218	7288.2	0.98 (0.91-1.05)
	Stage 1 HT	9,501	1,396	2063.6	1.08 (0.98-1.19)	5,493	7756.7	1.04 (0.99-1.09)
	Stage 2 HT	13,016	2,013	2243.8	1.18 (1.08-1.29)	7,961	8450.9	1.16 (1.11-1.21)

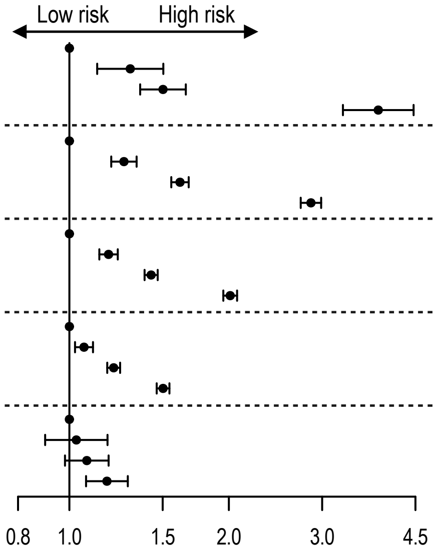

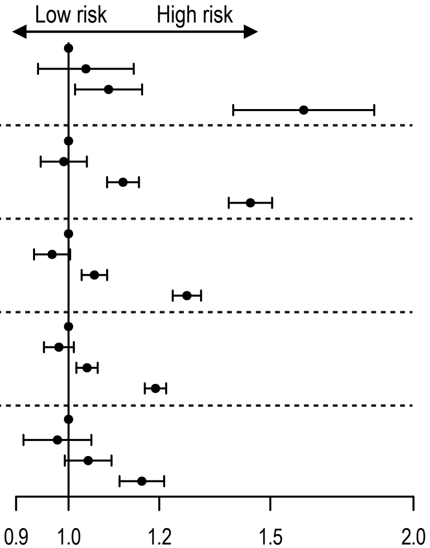

Figure S4. Sex- and age-specific CVD and mortality risks according to blood pressure group among persons not taking antihypertensive medication. Rates per 100,000 person-years. HRs adjusted for age, sex, household income, Charlson Comorbidity Index, use of glucose-lowering drugs, use of lipid-lowering drugs, smoking, drinking, exercise, body mass index, fasting glucose, and total cholesterol. BP, blood pressure; CI, confidence interval; CVD, cardiovascular disease; HR, hazard ratio; HT, hypertension.

Age, y	BP group	Persons	CVD hospitalization: Events	Rate*	HR (95% CI)	All-cause death: Events	Rate*	HR (95% CI)
20-34	Normal	1,937,241	2,952	15.4	1.00 (reference)	7,783	40.6	1.00 (reference)
	Elevated	457,332	1,055	23.4	1.13 (1.05-1.21)	2,239	49.6	1.00 (0.95-1.04)
	Stage 1 HT	1,371,451	4,414	32.7	1.39 (1.32-1.46)	7,585	56.1	1.07 (1.04-1.11)
	Stage 2 HT	313,283	2,747	89.1	2.79 (2.64-2.96)	2,676	86.6	1.47 (1.40-1.54)
35-49	Normal	2,175,833	14,262	66.3	1.00 (reference)	25,332	117.5	1.00 (reference)
	Elevated	588,708	5,370	92.4	1.22 (1.19-1.26)	7,643	131.1	1.04 (1.01-1.06)
	Stage 1 HT	1,891,527	23,507	126.2	1.51 (1.48-1.54)	28,894	154.5	1.14 (1.12-1.16)
	Stage 2 HT	655,503	15,844	247.5	2.55 (2.49-2.61)	15,459	239.2	1.60 (1.57-1.63)
50-64	Normal	875,337	19,114	224.6	1.00 (reference)	34,709	404.6	1.00 (reference)
	Elevated	307,662	8,319	278.9	1.15 (1.12-1.18)	12,859	426.7	1.02 (1.00-1.04)
	Stage 1 HT	1,014,746	33,663	343.8	1.35 (1.33-1.38)	47,577	479.8	1.09 (1.07-1.10)
	Stage 2 HT	518,297	26,416	536.4	1.93 (1.89-1.97)	33,453	665.7	1.40 (1.38-1.42)
65-79	Normal	180,966	13,841	862.4	1.00 (reference)	37,042	2247.9	1.00 (reference)
	Elevated	84,476	7,097	944.0	1.08 (1.05-1.12)	16,485	2129.1	0.98 (0.96-1.00)
	Stage 1 HT	277,968	26,256	1069.5	1.21 (1.18-1.23)	56,667	2232.2	1.03 (1.01-1.04)
	Stage 2 HT	213,587	25,796	1406.4	1.54 (1.51-1.57)	51,144	2668.0	1.21 (1.19-1.23)
80-94	Normal	8,284	1,043	1902.0	1.00 (reference)	5,452	9583.6	1.00 (reference)
	Elevated	3,889	525	1987.5	1.04 (0.94-1.16)	2,450	8889.7	0.98 (0.94-1.03)
	Stage 1 HT	15,508	2,146	2066.7	1.08 (1.00-1.17)	9,976	9190.7	1.01 (0.97-1.04)
	Stage 2 HT	17,245	2,641	2356.6	1.24 (1.15-1.33)	11,624	9892.2	1.13 (1.09-1.17)

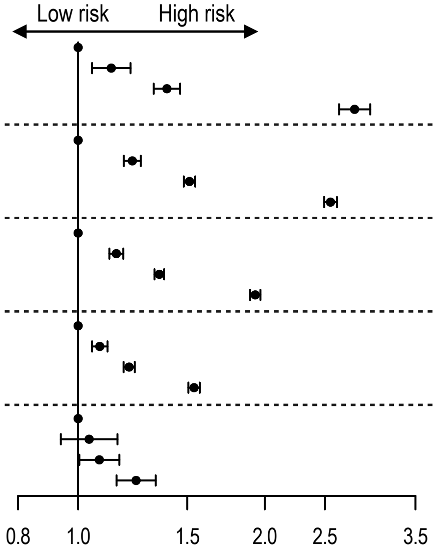

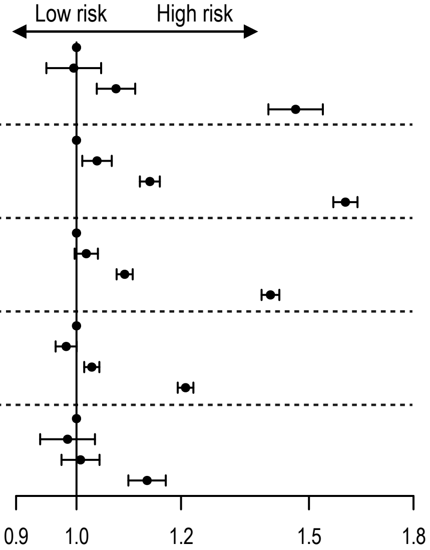

Figure S5. Age-specific CVD and mortality risks according to blood pressure group among persons who have not started antihypertensive medications up to one year of follow-up. Rates per 100,000 person-years. HRs adjusted for age, sex, household income, Charlson Comorbidity Index, use of glucose-lowering drugs, use of lipid-lowering drugs, smoking, drinking, exercise, body mass index, fasting glucose, and total cholesterol. BP, blood pressure; CI, confidence interval; CVD, cardiovascular disease; HR, hazard ratio; HT, hypertension.

Age, y	BP group	Persons	CVD hospitalization: Events	CVD hospitalization: Rate*	CVD hospitalization: HR (95% CI)	All-cause death: Events	All-cause death: Rate*	All-cause death: HR (95% CI)
20-34	Normal	1,941,381	2,978	15.5	1.00 (reference)	7,840	40.8	1.00 (reference)
	Elevated	458,756	1,066	23.5	1.13 (1.06-1.22)	2,263	49.9	1.00 (0.95-1.05)
	Stage 1 HT	1,378,744	4,509	33.2	1.41 (1.34-1.48)	7,698	56.6	1.08 (1.04-1.11)
	Stage 2 HT	361,794	3,611	101.5	3.08 (2.92-3.25)	3,274	91.7	1.53 (1.46-1.60)
35-49	Normal	2,192,925	14,591	67.3	1.00 (reference)	25,997	119.7	1.00 (reference)
	Elevated	595,950	5,556	94.5	1.24 (1.20-1.28)	7,868	133.3	1.03 (1.01-1.06)
	Stage 1 HT	1,934,551	24,515	128.8	1.54 (1.51-1.57)	30,135	157.5	1.14 (1.12-1.16)
	Stage 2 HT	1,171,007	30,989	271.2	2.69 (2.63-2.74)	28,569	247.5	1.54 (1.51-1.57)
50-64	Normal	897,269	20,121	230.9	1.00 (reference)	36,636	416.9	1.00 (reference)
	Elevated	319,636	8,944	288.9	1.17 (1.14-1.20)	13,709	438.2	1.01 (0.99-1.03)
	Stage 1 HT	1,077,967	36,930	355.4	1.38 (1.35-1.40)	51,984	493.8	1.09 (1.07-1.10)
	Stage 2 HT	1,620,496	87,702	569.7	1.99 (1.96-2.02)	102,289	650.4	1.33 (1.32-1.35)
65-79	Normal	192,162	15,279	900.6	1.00 (reference)	40,341	2313.4	1.00 (reference)
	Elevated	91,396	7,970	984.0	1.09 (1.06-1.12)	18,224	2181.9	0.97 (0.96-0.99)
	Stage 1 HT	311,521	30,482	1113.7	1.21 (1.19-1.24)	65,339	2305.3	1.03 (1.02-1.04)
	Stage 2 HT	871,168	112,670	1503.7	1.58 (1.56-1.61)	203,205	2587.6	1.19 (1.18-1.21)
80-94	Normal	9,094	1,170	1952.9	1.00 (reference)	6,013	9651.5	1.00 (reference)
	Elevated	4,347	612	2089.0	1.07 (0.97-1.18)	2,748	8954.1	0.97 (0.93-1.02)
	Stage 1 HT	17,844	2,569	2162.6	1.11 (1.03-1.19)	11,517	9253.2	1.00 (0.97-1.04)
	Stage 2 HT	60,529	10,417	2600.0	1.33 (1.25-1.42)	39,284	9276.6	1.10 (1.07-1.13)

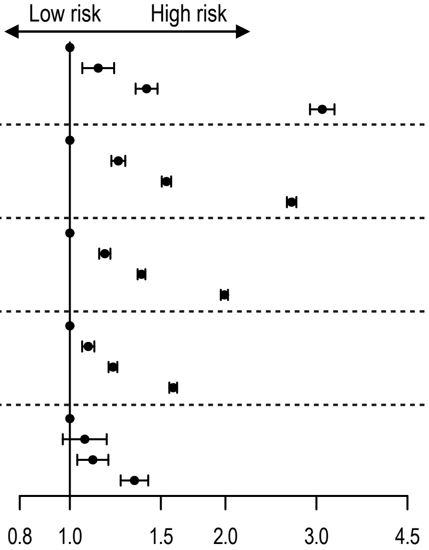

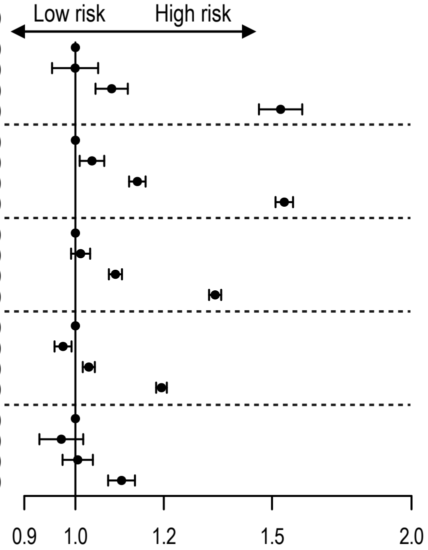

Figure S6. Age-specific CVD and mortality risks according to blood pressure group with stage 2 hypertension defined as systolic BP ≥140 mmHg or diastolic BP ≥90 mmHg or treated. Rates per 100,000 person-years. HRs adjusted for age, sex, household income, Charlson Comorbidity Index, use of glucose-lowering drugs, use of lipid-lowering drugs, smoking, drinking, exercise, body mass index, fasting glucose, and total cholesterol. BP, blood pressure; CI, confidence interval; CVD, cardiovascular disease; HR, hazard ratio; HT, hypertension.

Age, y	BP group	Persons	CVD hospitalization: Events	Rate*	HR (95% CI)	All-cause death: Events	Rate*	HR (95% CI)
20-34	Normal	1,947,809	3,031	15.7	1.00 (reference)	7,904	41.0	1.00 (reference)
	Elevated	461,139	1,096	24.1	1.14 (1.06-1.22)	2,292	50.3	1.00 (0.95-1.05)
	Stage 1 HT	1,392,299	4,663	34.0	1.41 (1.34-1.48)	7,831	57.0	1.08 (1.04-1.11)
	Stage 2 HT	339,428	3,374	101.1	3.01 (2.85-3.18)	3,048	91.0	1.51 (1.44-1.58)
35-49	Normal	2,249,322	15,872	71.4	1.00 (reference)	27,364	122.8	1.00 (reference)
	Elevated	624,848	6,261	101.6	1.25 (1.21-1.28)	8,496	137.3	1.03 (1.01-1.06)
	Stage 1 HT	2,098,842	28,735	139.2	1.54 (1.51-1.57)	33,724	162.5	1.13 (1.11-1.15)
	Stage 2 HT	921,421	24,783	275.8	2.60 (2.54-2.65)	22,985	253.2	1.58 (1.55-1.61)
50-64	Normal	1,015,542	25,706	261.1	1.00 (reference)	43,588	438.7	1.00 (reference)
	Elevated	397,100	12,597	328.4	1.16 (1.13-1.18)	18,089	465.9	1.01 (1.00-1.03)
	Stage 1 HT	1,441,198	55,275	398.8	1.35 (1.33-1.37)	72,259	513.8	1.07 (1.06-1.09)
	Stage 2 HT	1,061,528	60,119	597.9	1.86 (1.83-1.88)	70,682	687.2	1.36 (1.34-1.37)
65-79	Normal	253,239	22,861	1029.1	1.00 (reference)	54,914	2396.0	1.00 (reference)
	Elevated	138,820	13,518	1104.2	1.06 (1.04-1.08)	28,395	2240.7	0.97 (0.95-0.98)
	Stage 1 HT	511,822	55,823	1246.3	1.18 (1.16-1.20)	108,846	2336.7	1.02 (1.01-1.03)
	Stage 2 HT	562,366	74,199	1542.2	1.43 (1.41-1.45)	134,954	2672.7	1.17 (1.16-1.19)
80-94	Normal	12,845	1,804	2134.2	1.00 (reference)	8,459	9574.9	1.00 (reference)
	Elevated	6,974	1,062	2257.0	1.05 (0.98-1.14)	4,387	8853.9	0.97 (0.94-1.01)
	Stage 1 HT	29,996	4,692	2332.7	1.09 (1.03-1.15)	19,140	9041.6	1.00 (0.97-1.02)
	Stage 2 HT	41,999	7,210	2612.9	1.22 (1.16-1.29)	27,576	9464.9	1.09 (1.06-1.12)

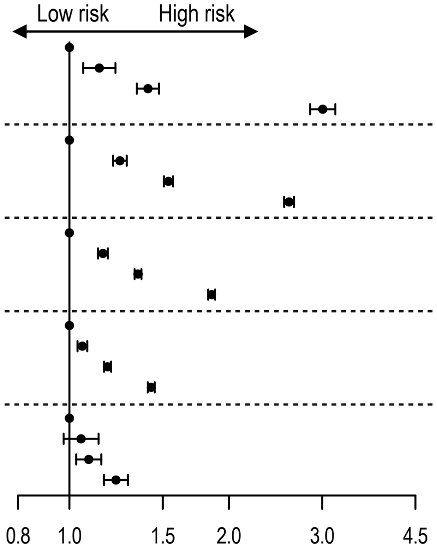

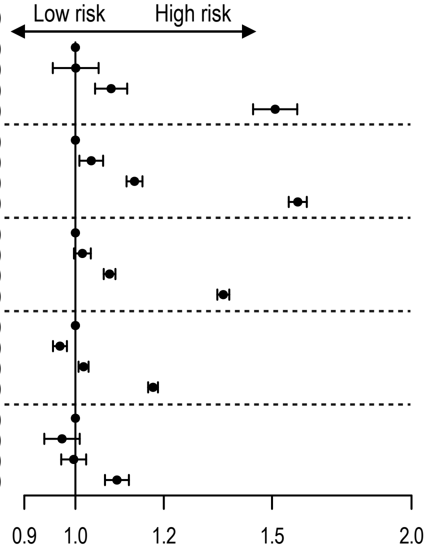

Figure S7. Age-specific CVD and mortality risks according to blood pressure group with or without antihypertensive medication. Rates per 100,000 person-years. HRs adjusted for age, sex, household income, Charlson Comorbidity Index, use of glucose-lowering drugs, use of lipid-lowering drugs, smoking, drinking, exercise, body mass index, fasting glucose, and total cholesterol. BP, blood pressure; CI, confidence interval; CVD, cardiovascular disease; HR, hazard ratio; HT, hypertension.

Age, y	BP group	Persons	CVD hospitalization Events	CVD hospitalization Rate*	CVD hospitalization HR (95% CI)	All-cause death Events	All-cause death Rate*	All-cause death HR (95% CI)
20-34	Normal	1,412,290	2,469	17.6	1.00 (reference)	5,589	39.8	1.00 (reference)
	Elevated	254,094	743	29.5	1.21 (1.12-1.32)	1,291	51.2	1.04 (0.98-1.10)
	Stage 1 HT	540,268	2,606	48.7	1.75 (1.65-1.86)	3,264	60.9	1.18 (1.13-1.24)
	Stage 2 HT	63,049	878	141.2	3.83 (3.52-4.17)	617	98.7	1.77 (1.62-1.93)
35-49	Normal	1,716,516	11,955	70.4	1.00 (reference)	18,365	107.8	1.00 (reference)
	Elevated	360,387	3,794	106.6	1.31 (1.26-1.35)	4,571	127.9	1.09 (1.05-1.12)
	Stage 1 HT	897,009	13,203	149.4	1.66 (1.62-1.70)	13,686	154.1	1.22 (1.20-1.25)
	Stage 2 HT	164,493	4,671	290.9	2.82 (2.72-2.92)	3,974	244.8	1.79 (1.72-1.85)
50-64	Normal	770,680	16,957	225.8	1.00 (reference)	27,461	362.7	1.00 (reference)
	Elevated	206,800	6,053	301.7	1.21 (1.18-1.25)	8,279	408.1	1.04 (1.02-1.07)
	Stage 1 HT	528,323	19,101	374.7	1.44 (1.41-1.47)	24,140	467.0	1.14 (1.12-1.16)
	Stage 2 HT	145,425	7,817	565.3	2.00 (1.94-2.05)	8,977	635.2	1.44 (1.41-1.48)
65-79	Normal	159,654	12,258	854.4	1.00 (reference)	29,557	2005.5	1.00 (reference)
	Elevated	59,767	5,095	947.6	1.09 (1.06-1.13)	10,636	1919.8	0.99 (0.96-1.01)
	Stage 1 HT	143,690	14,028	1097.2	1.26 (1.23-1.29)	27,445	2071.8	1.07 (1.05-1.09)
	Stage 2 HT	55,332	6,538	1353.2	1.52 (1.47-1.56)	11,886	2355.3	1.21 (1.18-1.24)
80-94	Normal	5,464	725	1909.1	1.00 (reference)	3,329	8421.2	1.00 (reference)
	Elevated	2,206	332	2159.6	1.13 (0.99-1.29)	1,321	8190.2	1.02 (0.95-1.08)
	Stage 1 HT	5,907	917	2246.9	1.18 (1.07-1.30)	3,632	8469.8	1.06 (1.01-1.11)
	Stage 2 HT	3,025	495	2421.1	1.28 (1.14-1.44)	1,929	8932.2	1.15 (1.09-1.22)

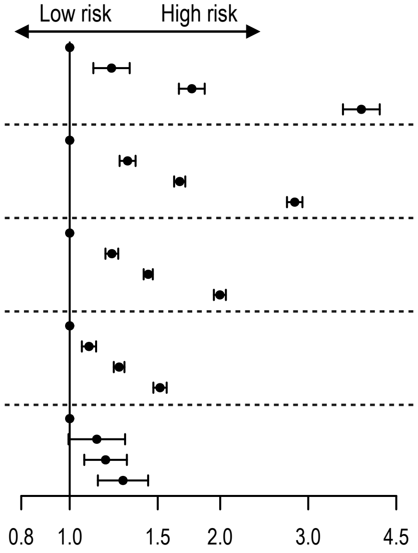

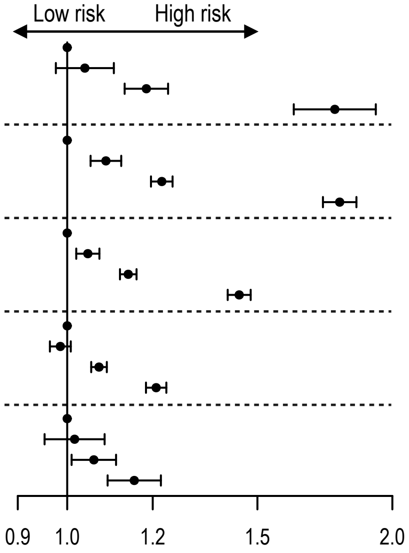

Figure S8. Age-specific CVD and mortality risks according to blood pressure group defined by measurements from two different examinations. Rates per 100,000 person-years. HRs adjusted for age, sex, household income, Charlson Comorbidity Index, use of glucose-lowering drugs, use of lipid-lowering drugs, smoking, drinking, exercise, body mass index, fasting glucose, and total cholesterol. BP, blood pressure; CI, confidence interval; CVD, cardiovascular disease; HR, hazard ratio; HT, hypertension.
